# BIS‐guided deep anesthesia decreases short‐term postoperative cognitive dysfunction and peripheral inflammation in elderly patients undergoing abdominal surgery

**DOI:** 10.1002/brb3.1238

**Published:** 2019-02-27

**Authors:** Chengxuan Quan, Jia Chen, Yuting Luo, Lei Zhou, Xi He, Yan Liao, Jing Chou, Qulian Guo, Alex F. Chen, Ouyang Wen

**Affiliations:** ^1^ Department of Anesthesia the Third Xiangya Hospital of Central South University Changsha China; ^2^ Department of Anesthesia Xiangya Hospital of Central South University Changsha China; ^3^ Department of Cardiology the Third Xiangya Hospital of Central South University Changsha China; ^4^ Department of Surgery University of Pittsburgh School of Medicine and Veterans Affairs Pittsburgh Healthcare System Pittsburgh Pennsylvania

**Keywords:** bispectral index (BIS), depth of anesthesia, postoperative cognitive dysfunction (POCD), total intravenous anesthesia

## Abstract

**Objectives:**

Postoperative cognitive dysfunction (POCD) is a common clinical complication, with an underlying pathophysiology linked to heightened levels of neuroinflammation. However, it requires clarification as to whether the depth of anesthesia modulates postoperative cognitive dysfunction. This study investigated the association between depth of anesthesia and POCD in elderly patients undergoing abdominal surgery.

**Methods:**

A total of 120 patients aged 60 years or older who were planned for abdominal surgery under total intravenous anesthesia were included in this study. The depth of anesthesia was guided by monitoring Bispectral Index (BIS) data. All study participants completed a battery of nine neuropsychological tests before surgery and at 7 days and 3 months after surgery. POCD was calculated by using the reliable change index. Plasma concentration of C‐reactive protein (CRP), interleukin (IL)‐1β, IL‐10, S‐100β, and norepinephrine (NE) were measured.

**Results:**

The incidence of POCD at 7 days after surgery in the deep anesthesia group was 19.2% (10/52), which was significantly lower (*p* = 0.032) than the light anesthesia group 39.6% (21/53). The depth of anesthesia had no effect on POCD at 3 months after surgery (10.3% vs 14.6%, respectively, *p* = 0.558). Similarly, plasma levels of CRP and IL‐1β in deep anesthesia group were lower than that in light anesthesia group at 7 days after surgery (*p* < 0.05), but not at 3 months after surgery (*p* > 0.05). There were no significant differences in the plasma concentration of IL‐10, S‐100β, and NE between the groups (*p* > 0.05).

**Conclusions:**

Deep anesthesia under total intravenous anesthesia could decrease the occurrence of short‐term POCD and inhibit postoperative peripheral inflammation in elderly patients undergoing abdominal surgery, compared with light anesthesia.

## 1 INTRODUCTION

Postoperative cognitive dysfunction (POCD) is a common clinical complication, with impacts on a wide array of cognitive domains, including attention, memory, executive function, and information processing speed. POCD is also associated with longer hospital admissions, increased mortality, and decreased longer term quality of life (Moller et al., 1998; Monk et al., 2008; Newman, Grocott et al., 2001; Newman, Kirchner et al., 2001). While reversible, POCD affects up to 16%–40% of middle‐aged and elderly patients at 7 days after surgery and 10%–13% of elderly patients at 3 months or longer after surgery (Moller et al., 1998; Monk et al., 2008; Rundshagen, 2014; Steinmetz, Christensen, Lund, Lohse, & Rasmussen, 2009). The aetiology of POCD remains unclear, being thought more generally to be related to a combination of surgery, anesthesia, and patient‐related factors (Rundshagen, 2014; Silbert et al., 2015; Van Harten, Scheeren, & Absalom, 2012).

Overexposure to anesthetics can have side effects (Alan, Mutch, Renée, El‐Gabalawy, & Ruth Graham, 2018), with the monitoring anesthesia depth contributing to reduced anesthetic exposure and consequently decreasing the risk of cognitive decline after surgery (Chan, Cheng, Lee, & Gin, 2013). However, as to how the depth of anesthesia modulates POCD requires clarification. Previous data indicate that deeper levels of combined intravenous inhalation anesthesia, as measured by the Bispectral Index (BIS), was associated with better cognitive function at 4–6 weeks after noncardiac surgery in patients older than 50 years (Farag, Chelune, Schubert, & Mascha, 2006). Another study reported that deeper total intravenous anesthesia, as guided by BIS, could decrease the incidence of POCD at 5 days after microvascular decompression in patients aged 28–67 years (An et al., 2011). One more study also found that deeper depth of anesthesia guided by AAI (AEP index) decreased the incidence of POCD, defined as MMT (the mini‐mental test) scores below 25, at 1 day after ophthalmic surgery under general anesthesia (Jildenstal, Hallen, Rawal, Gupta, & Berggren, 2011). However, there have been some mixed results, with one study showing no significant association of general anesthesia depth and POCD at 7 days following elective noncardiac surgery in elderly patients (Steinmetz & Funder, 2010), whilst another study found no association of general anesthesia depth and POCD at 7 days and 3 months after surgery in elderly patients (Radtke et al., 2013). However, a number of factors complicate the interpretation of previous data, including anesthetic depth beyond the planned target range and combinations too many of commonly prescribed drugs (Farag et al., 2006), lack of age stratification (An et al., 2011), small sample sizes (Steinmetz & Funder, 2010), and a lack of group randomization (Radtke et al., 2013). Clearly more carefully collected data are required to better clarify the association of anesthesia depth and POCD, especially for elderly patients.

This study investigated the incidence of POCD at 7 days and 3 months postoperatively and the association of POCD with anesthesia depth, as defined by BIS under total intravenous anesthesia. This study also investigated some of the relevant biological underpinnings of POCD, and the implications that this may have for clinical management and future investigations.

## 2 MATERIALS AND METHODS

### 2.1 Participants and study design

This is a randomized parallel controlled clinical trial investigating the association with anesthesia depth and POCD incidence at 7 days and 3 months following abdominal surgery in elderly patients (≥60 years). After obtaining IRB approval (2014‐S008, Human Research Ethics Committee, the Third Xiangya Hospital of Central South University, Changsha, China) and informed consent (120 ASA I–II), all 60 years or older patients scheduled to undergo abdominal surgery from February 2014 to February 2016 were randomly divided into two groups: Deep group (deep anesthesia group with BIS target 30–45) (*n* = 60) and Light group (light anesthesia group with BIS target 45–60) (*n* = 60). Exclusion criteria: patients with a preexisting neurological or clinically evident neurovascular diseases; mini‐mental state examination (MMSE) score <23 before surgery; anticipated difficulty with neuropsychological assessment; associated medical problems that may lead to significant complications; blindness, deafness, Chinese not being first language; drug or alcohol abuse; prior surgery; severe perioperative complications.

### 2.2 Neuropsychology tests

Neuropsychological testing, consisted of a battery of nine neuropsychological tests, were administered at baseline (1 day before surgery) and at 7 days, and 3 months after surgery. An experienced psychometrician who was blinded to anesthesia management carried out the assessments. The neuropsychological tests included: (1) the Mental Control and (2, 3) Digit Span (forward and backward) subtests of the Wechsler Memory Scale (Chinese edition, Central South University) ‐measures of attention and concentration, with high scores indicating better function; (4) the Visual Retention, and (5) Paired Associate Verbal Learning subtests of the Wechsler Memory Scale (Chinese edition, Central South University)‐measures of figurative memory and verbal learning/memory, with high scores indicating better function; (6) the Digit Symbol subtest of the Wechsler Adult Intelligence Scale‐Revised (Chinese edition, Central South University)‐ a measure of psychomotor speed, with a high score indicating better function; (7) the Halstead‐Reitan Trail Making Test (Part A)‐a measure of hand‐eye coordination, attention, and concentration, with a low score indicating better function; and (8, 9) the Grooved Pegboard Test (favored and unfavored hand)‐a measure of manual dexterity, with a low score indicating better function. We applied the definition proposed by Newman for postoperative cognitive deficits (Newman, 1995; Newman, Grocott et al., 2001; Newman, Kirchner et al., 2001). The standard deviation for each test was computed from all of the preoperative scores. An individual whose postoperative performance deteriorated by 1 or more standard deviations on 2 or more tests was classified as having POCD.

### 2.3 Anesthesia protocol and management

Total intravenous anesthesia was used during the operation, while induction with propofol, sufentanil, cis‐atracurium, and maintenance with propofol and remifentanil. The anesthetic depth was titrated by using propofol to achieve the desired BIS target ranges in each group. Sufentanil and remifentanil were used only to provide background analgesia, not to adjust depth of anesthesia. The anesthesia depth in the Deep group was determined by BIS values ranging from 30–45; in the Light group, the anesthesia depth was determined by BIS values ranging from 45 to 60. In this way, BIS values would be maintained at no more than five units outside the target range for <5 min. The choice of BIS targets for Deep and Light groups is based on previous studies. Two studies (An et al., 2011; Farag et al., 2006) defined lighter anesthesia as being a BIS target of 50–60 and deeper anesthesia as BIS target of 30–40. The underlying large‐scale randomize study (Short et al., 2015) which would recruite 6,500 patients with 40 to 50 centers internationally defined “light” anesthesia as a BIS target of 50 and “deep” anesthesia as a BIS target of 35. However, as BIS values usually fluctuate quickly, it is difficult to maintain BIS values in a narrow range. As a BIS value of 45 is generally regarded as the dividing line between “light” anesthesia and “deep” anesthesia, BIS values ranging from 30 to 45 are treated here as “deep” anesthesia and 45–60 as “light” anesthesia. This allowed for the target BIS values to be maintained for more than 80% of the total time from the induction to the cessation of anesthesia, as reported previously (An et al., 2011).

There were no restrictions on the use of muscle relaxants. Neuromuscular blocking agents were used by instructions at the end of surgery. Postoperative analgesia was provided as a standard practice in this department, and depended on surgery type and duration. Arterial blood pressure (BP) was controlled to within 25% of the baseline (before the day of surgery) systolic value and heart rate (HR) was controlled to 50–90 bpm as appropriate. If the BP went outside this target range, it was increased with phenylephrine or decreased with nitroglycerin. HR was controlled by using isoprenaline or esmolol. Mean arterial pressure (MAP) and HR were recorded at the following time points: before surgery, tracheal intubation, skin incision, maximum trauma, end of surgery, extubation, and back to ward. A blinded observer in the PACU (postanesthesia care unit) monitored the recovery indexes, such as agitation, extubation time, ARS (Aldrete Recovery Score), RASS (Richmond Agitation‐Sedation Scale), and VAS (Visual Analogue Score) scores as well as postoperative nausea and vomiting (PONV). All patients were discharged to the ward following a 2 hr stay in the PACU, with the modified Aldrete score ≥9, as well as all discomfort being appropriately controlled. Postoperative complications and hospital stay and cost were also recorded.

### 2.4 Plasma C‐reactive protein (CRP), interleukin (IL)‐1β, IL‐10, S‐100β, and norepinephrine (NE) measurement

Blood samples were drawn in EDTA‐containing tubes at the following time points: before surgery, end of surgery, and postsurgery days 1, 2, and 7 as well as 3 months after surgery. The samples were immediately transferred to the biomedical laboratory and centrifuged for 10 min at 3000‐g. Plasma was removed and stored at −80°C until analysis. The plasma concentration of CRP, IL‐1β, IL‐10, S‐100β were measured, by using an ELISA Immulite 1000 kit (IL‐1β, IL‐10, S‐100β) or turbidimetric inhibition immuno assay (CRP). Each serum sample was blindly analyzed in duplicate and the result was expressed as the mean of two measurements. The perioperative plasma concentration of NE was also measured by ELISA Immulite 1000 kit at the same time points when patients’ MAP and HR were recorded, as noted previously.

### 2.5 Statistics

Statistical analyses were performed using the SPSS statistical package (version 18.0; SPSS Inc., Chicago, IL, USA). Group comparisons were made using independent *t* tests for continuous variables, the Mann–Whitney *U* test for ranked data, and chi‐square or Fisher exact test for dichotomous data. Correlation analyses were performed using Spearman's rank correlation. The Wald method was used to compute a 95% confidence interval for a proportion. Continuous data were reported as medians (5%–95% percentiles), mean (SD), and proportions indicated by percentages (%). Values of *p* < 0.05, two‐tailed, were considered statistically significant in all studies.

## 3 RESULTS

### 3.1 Enrolled patients

**Figure 1:**
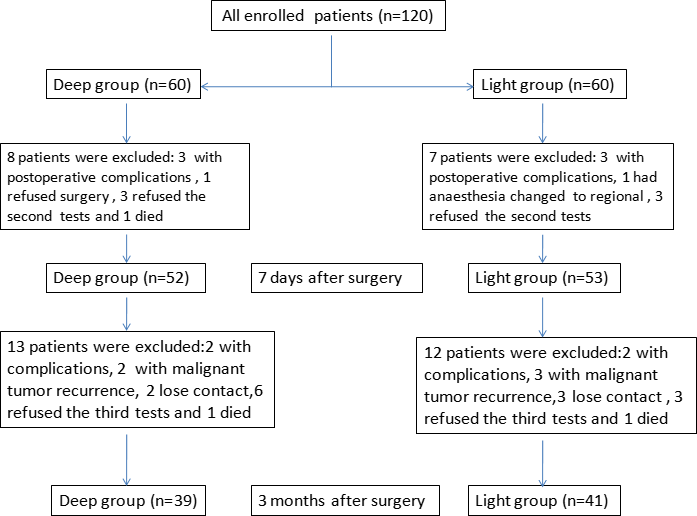
Drop‐out and follow‐up information of the enrolled patients

One hundred and five of the 120 patients completed both preoperative and 7 days postoperative neuropsychological testing. Eight patients dropped out from the Deep group and seven patients dropped out from the Light group at 7 days after surgery. Eighty patients completed postoperative neuropsychological testing at 3 months after surgery. Of the dropouts at 3 months, 13 were from the Deep group and 12 from the Light group. The reasons for drop‐out are shown in Figure 1. There was no statistically significant difference between the two groups with regard to the number of dropouts (Figure 1, *p* > 0.05).

### 3.2 Patients characteristics and intraoperative data

Patients characteristics such as age, gender, body mass index (BMI), education, and MMSE before surgery were similar between groups (Table 1, *p* > 0.05). There were also no substantive between‐group differences in intraoperative data except for propofol dosage (Table 1, *p* < 0.05). BIS values in the two groups were controlled in target range, and the difference in BIS values between groups from the completion of intubation to the completion of suturing were significant (Table 1, *p* < 0.05).

**Table 1:** Patients characteristics and intraoperative data

Characteristics	Deep group	Light group	*p* values
Age (y)	65.6 ± 3.77	63.9 ± 4.56	0.568
Gender (M/F)	34/26	32/28	0.733
BMI (kg/m^2^)	22.3 ± 3.4	21.4 ± 3.0	0.258
Education (y)	7.3 ± 2.6	8.2 ± 3.3	0.225
MMSE before surgery	26.6 ± 22.0	25.8 ± 2.9	0.294
Benign/Malignant cases	26/34	25/35	0.639
Anesthesia duration (min)	184.2 ± 47.9	193.3 ± 64.0	0.598
Surgery Duration (min)	177.5 ± 52.3	186.6 ± 74.1	0.608
Propofol (mg)	1308 ± 398	1024 ± 361	0.021^a^
Remifentanil (mg)	1.69 ± 0.70	1.86 ± 0.73	0.456
Sulfentanyl (μg)	73.89 ± 21.73	76.11 ± 18.44	0.696
Cis‐atracurium (mg)	21.28 ± 7.35	23.33 ± 5.28	0.348
Blood loss (ml)	198.4 ± 104.4	215.4 ± 125.5	0.579
Blood transfusion rate	9.6%(5/52)	13.2%(7/53)	0.567
Fluid infusion volum (ml)	2636 ± 620	2574 ± 769	0.750
Mean BIS values	39.2 ± 5.8	52.7 ± 6.2	<0.001^a^
% Avg BIS >60	2.2	5.7	
% Avg BIS 45–60	12.5	84.6	
% Avg BIS 30–45	85.3	9.7	
% Avg BIS < 30	*N* (almost)	*N*	

Notes.

Data were expressed as mean (*SD*) except for categorical data as number and percentage; *p*‐values with respect to chi‐squared test, Mann–Whitney *U*‐test, or Student *t*‐test, respectively, for independent variables.

BMI, body mass index; MMSE, mini‐mental state examination; % Avg BIS, percentage of BIS values in relation to total BIS values.

a *p* < 0.05.

### 3.3 Perioperative hemodynamics and recovery indexes

**Figure 2:**
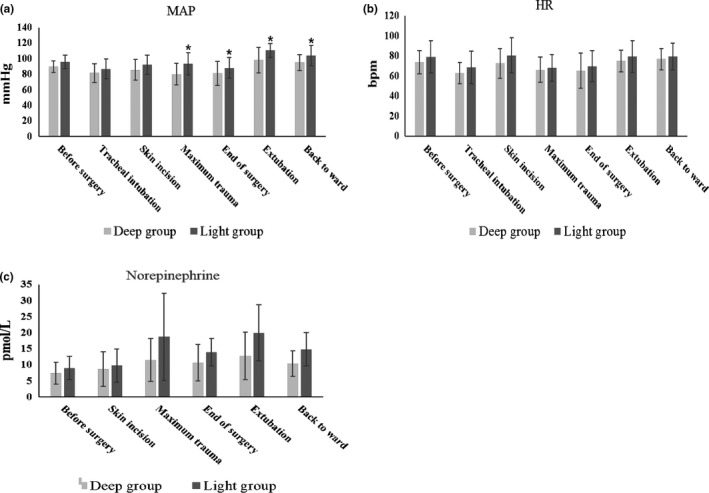
Perioperative MAP, HR and plasma level of Norepinephrine. MAP, mean arterial pressure; HR, heart rate. *p*‐values with respect to Student *t* test. *p < 0.05

The patients’ MAP in the Light group was higher than that in the Deep group at the time points of maximum trauma, end of surgery, extubation, and back to ward (Figure 2a, *p* < 0.05). There were no significant differences in HR between the two groups (Figure 2b, *p* > 0.05). There were also no significant difference at the concentration of plasma NE between groups (Figure 2c, *p* > 0.05). The extubation time in Deep group was significantly longer than that of the Light group (Table 2, *p* < 0.05). There were no significant differences in other recovery indexes, such as agitation, ARS, RASS, and VAS scores (Table 2, *p* > 0.05).

**Table 2:** Recovery indexes in two groups

Characteristics	Deep group	Light group	*p* values
Extubation time(min)	27.5 ± 11.9	18.7 ± 8.5	0.011^a^
ARS 1 hr after surgery	7.7 ± 1.8	8.7 ± 0.8	0.066
RASS 1 hr after surgery	0.1 ± 1.0	0.2 ± 0.7	0.819
VAS 1 hr after surgery	3.8 ± 2.4	4.7 ± 1.1	0.286
ARS 2 hr after surgery	8.9 ± 1.2	8.8 ± 1.4	0.786
RASS 2 hr after surgery	0.2 ± 0.4	0.2 ± 0.3	0.165
VAS 2 hr after surgery	4.4 ± 1.5	4.2 ± 1.4	0.817
Postoperative agitation	6/52 (11.5%)	5/53 (9.4%)	0.761

Notes.

Data were expressed as mean (*SD*) except for categorical data as number and percentage; *p*‐values with respect to chi‐squared test, Mann–Whitney *U*‐test, or Student *t*‐test, respectively, for independent variables.

ARS, Aldrete Recovery Score; VAS, Visual Analogue Score; RASS, Richmond Agitation‐Sedation Scale.

a *p* < 0.05.

### 3.4 The incidence of POCD and postoperative complications

The incidence of POCD at 7 days after surgery was 19.2% (10/52) in Deep group and 39.6% (21/53) in Light group, being significantly lower in the Deep group (Table 3, *p* = 0.032). However, the different depths of anesthesia had no effect on POCD incidence at 3 months after surgery (10.3% in deep anesthesia group vs. 14.6% in light anesthesia group, Table 3, *p* = 0.558). All patients’ neuropsychological tests are shown in Table 4. The complications after surgery are shown in Table 5 as well as hospital stay duration and cost (*p* > 0.05).

**Table 3:** The incidence of POCD at 7 days and 3 months after surgery

	7 days after surgery		3 months after surgery	
	POCD	Incidence	POCD	Incidence
Deep group	10/52	19.2%	4/39	10.3%
Light group	21/53	39.6%	6/41	14.6%
*p* values	0.032^a^		0.558	

Note.

*p*‐values with respect to chi‐squared test.

a *p* < 0.05.

**Table 4:** Group mean raw scores for all test subscales at the preoperative assessment (*M* ± *SD*)

Items	Deep group			Light group		
	Baseline	7 days	3 months	Baseline	7 days	3 months
Mental control	80.4 ± 16.7	71.5 ± 19.5	85.1 ± 8.4	78.2 ± 15.5	78.3 ± 21.7	85.5 ± 15.2
Visional rational	9.8 ± 2.6	7.8 ± 2.9	11 ± 1.7	9.0 ± 3.1	9.2 ± 2.4	10 ± 2.5
Paired associate verbal learning	10.4 ± 4.0	10.5 ± 4.4	13.9 ± 4.3	10.4 ± 4.4	11.1 ± 3.8	11.8 ± 3.7
Digit span forward	6.6 ± 1.3	6.8 ± 1.5	6.9 ± 1.2	6.9 ± 1.6	7.3 ± 1.3	7.3 ± 1.0
Digit span backward	3.2 ± 0.8	3.2 ± 1.0	3.3 ± 1.3	3.2 ± 0.8	3.2 ± 1.0	3.4 ± 0.8
Digit symbol	21.5 ± 12.3	18.6 ± 10.3	27.4 ± 15.4	20.4 ± 8.8	21.7 ± 10.7	28.1 ± 10.5
Trails A	184.5 ± 81.8	211.9 ± 77.9	163.6 ± 86.6	192.3 ± 85.3	180.7 ± 72.4	142.6 ± 39.3
Pegboard favored hand	115.8 ± 36.8	121.3 ± 33.7	83.8 ± 18.2	109.2 ± 34.1	117.6 ± 38.6	89.4 ± 20.5
Pegboard unfavored hand	120.8 ± 39.8	129.1 ± 37.3	91.3 ± 20.4	125.3 ± 41.1	132.4 ± 51.7	100.9 ± 40.1

Notes.

The standard deviation for each test was computed from all of the preoperative scores. An individual whose postoperative performance deteriorated by 1 or more standard deviations on 2 or more tests was classified as having experienced POCD.

**Table 5:** Postoperative complications and hospital stay duration and cost in two groups

Characteristics	Deep group	Light group
Hospital stay (day)	20 ± 5	18 ± 5
Hospital cost (CNY)	59,360 ± 16,008	53,774 ± 14,280
*Complications*		
First 7 days after surgery		
Pulmonary infection	7	8
Incision infection	3	2
Postoperative bleeding	2	0
Cricoarytenoid joint dislocation	0	2
ARDS	1	0
Intraoperative awareness	0	1
Pain (opioid need cases)	7 (2)	9 (4)
PONV	6	7
Death	1	0
3 months after surgery		
Incision infection	2	2
Pain (opioid need cases)	2 (0)	3 (0)
Rehospitalization	4	5
Death	1	1

Notes.

*p*‐values between groups in hospital stay duration and cost are 0.150 and 0.192, respectively. The death case in Deep group during first 7 days after surgery was the result of massive hemorrhage. The death cases 3 months after surgery in Deep and Light groups were the results of malignant tumor recurrence.

### 3.5 Plasma concentration of CRP, IL‐1β, IL‐10, and S‐100β

**Figure 3:**
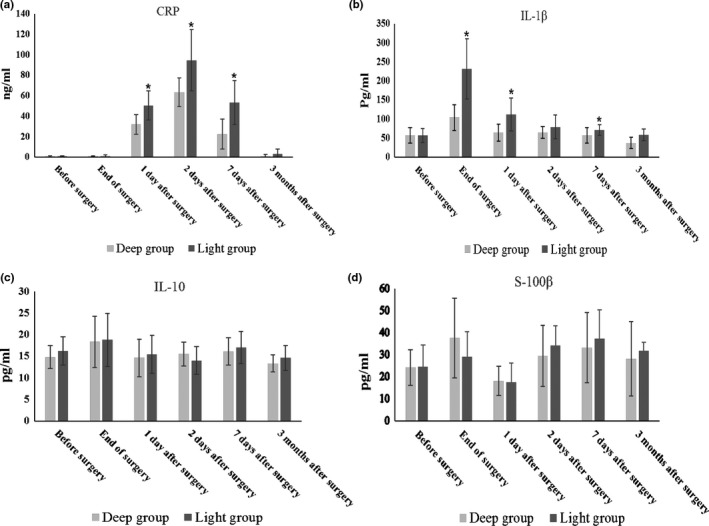
The plasma concentration of CRP, IL‐1β, IL‐10, and S‐100β. *p*‐values with respect to Student *t* test. *p < 0.05

The plasma concentration of CRP and IL‐1β ascended after surgery and decreased to baseline levels at 3 months follow‐up. Plasma levels of CRP and IL‐1β in the Deep versus Light groups were significantly lower at 7 days after surgery (Figure 3a,b, *p* < 0.05), but not at 3 months follow‐up (Figure 3a,b, *p* > 0.05). There were no significant difference in the plasma concentration of IL‐10 and S‐100β between the two groups at any time point (Figure 3c,d, *p* > 0.05).

## 4 DISCUSSION

This study is randomized parallel controlled clinical trial investigating how anesthesia depth may modulate POCD and some of the possible pathophysiological processes involved. It also attempts to overcome some of the design deficits in previous trials and better clarify the mixed results evident in prior publications in this area (An et al., 2011; Farag et al., 2006; Jildenstal et al., 2011; Radtke et al., 2013; Steinmetz & Funder, 2010). The first major finding in this study shows the incidence of POCD at 7 days postsurgery to be significantly lower in patients who underwent deep anesthesia, with incidence of POCD at 7 days postsurgery in Deep versus Light anesthesia groups being 19.2% (10/52) versus 39.6% (21/53) (*p* = 0.032). No significant difference in POCD incidence was evident between groups at 3 months postsurgery (10.3% vs. 14.6%, *p* = 0.558). Such data support a general trend evident in previous studies in this area (An et al., 2011; Farag et al., 2006; Jildenstal et al., 2011), whilst improving on the design protocol limitations, as noted in the introduction above.

As previous work, both clinically and preclinically, indicates that inflammation plays an important role in the pathophysiological changes underpinning POCD and cognitive deficits more widely (Cibelli et al., 2010; Hennessy et al., 2017; Heyer, Mergeche, Bruce, & Sander Connolly, 2013; Palta et al., 2015). This study also investigated the changes occurring in CRP, IL‐1β, IL‐10, and S100‐β. The second major finding of this current investigation indicates that plasma CRP and IL‐1β levels are decreased in the Deep versus Light groups at 7 days postsurgery. There were no significant differences between groups at the 3‐month follow‐up. Such data would suggest a relevant inverse covariance of CRP and IL‐1β with POCD, which is supportive of some previous data on the role of systemic inflammatory processes in the biological underpinnings of POCD (Jildenstål, Hallén, Rawal, Berggren, & Jakobsson, 2014).

This study also found no significant changes in the levels of NE, IL‐10, and S100‐β between groups. However, it should be noted that NE levels showed a trend increase in the Light group, which may suggest heightened stress responses contributing to the higher level of inflammation evident in this group. The present results may give some indication of this occurring as the patients’ MAP in Light group was higher versus the Deep group, at the time points of maximum trauma, end of surgery, extubation, and return to ward. As such, a heavier stress response to surgery in the Light group, characterized by a higher MAP combined with a trend faster HR, may increase the release of inflammatory factors, as reported previously (Vlachakis et al., 2017). There were no significant differences between groups at any time point regarding levels of the anti‐inflammatory cytokine, IL‐10, which has been shown to positively modulate cognitive functions (Kiyota et al., 2012; Xiu et al., 2016). This could suggest that levels of anti‐inflammatory responses are not significant determinants of POCD pathophysiology. Likewise, there were no significant differences in the levels of plasma S‐100β, which is often taken as an indicant of increased blood‐brain barrier (BBB) permeability in neurodegenerative diseases and traumatic brain injury as well as traumatic surgery in aged POCD rats (He et al., 2012). This likely indicates that the depth of anesthesia alone may not be a major contributor to increased BBB permeability during surgery.

It requires clarification as to how the increase in proinflammatory cytokines associated with a heightened incidence of POCD in the Light group. Via an increase in the activity of the kynurenine pathway may be a competitive explanation. It is likely that the increase in IL‐1β evident in this study is associated with a concurrent increase in IL‐18, given that both are cleaved to their active forms by caspase‐1 following inflammasome activation. Both IL‐1β and IL‐18, as with IL‐6 and TNF‐α, would be expected to increase indoleamine 2,3‐dioxygenase (IDO) and therefore increase kynurenine pathway products. Many kynurenine pathway products modulate neuronal and glia activity, with consequences for cognition, including levels of neurogenesis (Anderson & Maes, 2014; Zunszain et al., 2012). This may give some overlaps with the pathophysiology of depression (Anderson, 2018; Duan et al., 2018), which may be intimately linked to deficits in cognitive functioning (Ancelin et al., 2001). Interestingly, there is some data to suggest that increased levels of depression may be associated with POCD risk (Tzimas, Andritsos, Arnaoutoglou, Papathanakos, & Papadopoulos, 2016). This requires clarification in future studies.

The present results also require further investigation as to the source of increased proinflammatory activity. Recent preclinical work indicates that anesthetics can alter the diversity of the gut microbiome (Serbanescu et al., 2019), suggesting that a consequent increase in gut permeability may contribute to the raised levels of proinflammatory cytokines. In this context, it may also be of note that prebiotics have been shown to decrease the incidence of POCD in preclinical models (Yang, Wang, Wu, & Jiao, 2018). Heightened levels of postoperative proinflammatory cytokines can also prevent the synthesis of pineal melatonin (Pontes, Cardoso, Carneiro‐Sampaio, & Markus, 2007), suggesting that the raised cytokine levels evident in the Light group, may be associated with alterations in circadian regulation, as well as the antioxidant, anti‐inflammatory, and mitochondria‐optimizing effects of melatonin (Polyakova et al., 2018). The presurgical administration of melatonin can decrease POCD incidence in elderly patients (Fan, Yuan, Ji, Yang, & Gao, 2017).

This study has a number of limitations. The main limitations inherent in the design and findings were that long‐term recovery indexes, especially the association between long‐term mortality and depth of anesthesia, were not observed. Recently B‐Unaware Study Group (Kertai et al., 2010) proposed that increased intermediate term mortality is associated with cumulative time of low BIS (BIS<45) values in patients after noncardiac surgery. Sessler (Sessler et al., 2012) later reported A Triple Low of MAP, BIS, and MAC was a strong and highly statistically significant predictor for mortality. However, this has been the subject of some controversial debate (Kertai, White, & Gan, 2014; Papachristofi, Mackay, Powell, Nashef, & Sharples, 2014; Willingham et al., 2015). In this study, two patients died in Deep group and one in Light group, with none of these obviously associated with the depth of anesthesia (massive hemorrhage within 7 days and two cases of malignant tumor recurrence). No indicants of surgical complications differed between the two groups, with the exception of a longer extubation time in the Deep anesthesia group. Other limitations include no measures of sleep/circadian/melatonin dysregulation, mood, and indicants of gut permeability, such as circulating lipopolysaccharide (LPS) levels. As any impacts of LPS on cognition may be inhibited by vagal nerve stimulation in preclinical models and alterations in vagal functioning are evident under anesthesia (Sinclair, Téllez, Tapia, Ulkatan, & Deletis, 2017), it will be important to look at such indices in future research. Future research should clarify the relevance of such factors to the pathophysiology of POCD, as they may have immediately applicable implications for the clinical management and prevention of POCD.

In conclusion, the data of this study clearly show that deep anesthesia, as defined by a BIS target 30–45, versus light anesthesia (BIS target 45–60), was associated with a reduced incidence of short‐term POCD and decreased release of inflammatory cytokines in elderly patients undergoing abdominal surgery. The data also hint at the upstream and downstream mediators of proinflammatory processes in POCD, which should lead to further research with direct implications for the understanding and clinical management of this common postoperative complication.

## CONFLICT OF INTEREST

The authors declare no conflicts of interest.
